# Sperm Cell Driven Microrobots—Emerging Opportunities and Challenges for Biologically Inspired Robotic Design

**DOI:** 10.3390/mi11040448

**Published:** 2020-04-23

**Authors:** Ajay Vikram Singh, Mohammad Hasan Dad Ansari, Mihir Mahajan, Shubhangi Srivastava, Shubham Kashyap, Prajjwal Dwivedi, Vaibhav Pandit, Uma Katha

**Affiliations:** 1 Physical Intelligence Department, Max Planck Institute for Intelligent Systems, 70569 Stuttgart, Germany; 2 Department of Chemical and Product Safety, German Federal Institute for Risk Assessment (BfR), Max-Dohrn-Strasse 8-10, 10589 Berlin, Germany; 3 The BioRobotics Institute, Scuola Superiore Sant’Anna, Via Rinaldo Piaggio 34, 56025 Pontedera, Italy; hasan.mohammad@santannapisa.it; 4 Department of Excellence in Robotics & AI, Scuola Superiore Sant’Anna, Via Rinaldo Piaggio 34, 56025 Pontedera, Italy; 5 Königin-Olga-Stift Gymnasium, Johannesstraße 18, 70176 Stuttgart, Germany; mihir.mahajan2002@gmail.com; 6 Department of Zoology, Institute of Science, Banaras Hindu University, Varanasi 221005, India; shubhangibhu123@rediffmail.com; 7 Dr. A.P.J. Abdul Kalam Technical University, Lucknow 226031, India; shubham33103@gmail.com; 8 Department of Physics, Shri Ramswaroop Memorial University, Lucknow 226007, India; prajjwaldwivedi18@gmail.com; 9 Dynex Technologies, 14340 Sullyfield Circle, Chantilly, VA 20151-1621 USA; vaibhav.a.pandit@gmail.com; 10 BioPharma Division, GALAB Laboratories GmbH, 21029 Hamburg, Germany; uma.reddyx@gmail.com

**Keywords:** biohybrid, spermbot, microrobot, in vitro fertilization (IVF), targeted drug delivery

## Abstract

With the advent of small-scale robotics, several exciting new applications like Targeted Drug Delivery, single cell manipulation and so forth, are being discussed. However, some challenges remain to be overcome before any such technology becomes medically usable; among which propulsion and biocompatibility are the main challenges. Propulsion at micro-scale where the Reynolds number is very low is difficult. To overcome this, nature has developed flagella which have evolved over millions of years to work as a micromotor. Among the microscopic cells that exhibit this mode of propulsion, sperm cells are considered to be fast paced. Here, we give a brief review of the state-of-the-art of Spermbots—a new class of microrobots created by coupling sperm cells to mechanical loads. Spermbots utilize the flagellar movement of the sperm cells for propulsion and as such do not require any toxic fuel in their environment. They are also naturally biocompatible and show considerable speed of motion thereby giving us an option to overcome the two challenges of propulsion and biocompatibility. The coupling mechanisms of physical load to the sperm cells are discussed along with the advantages and challenges associated with the spermbot. A few most promising applications of spermbots are also discussed in detail. A brief discussion of the future outlook of this extremely promising category of microrobots is given at the end.

## 1. Introduction

**Figure 1:**
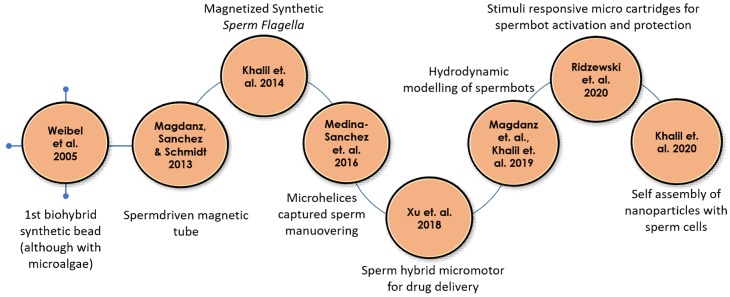
Timeline for biohybrid sperm development, starting with the first biohybrid synthetic bead.

Nature becomes an excellent teacher when we seek solutions to complex problems that cannot be solved using contemporary engineering principles [1]. At times, instead of mimicking nature, it can be directly used to our advantage [2]. When bioengineers translate fundamental biological principles into intricate designs of micro/nanorobots for Targeted Drug Delivery (TDD), they adopt the functionality of living systems. Soft-robotic systems have been introduced for reducing the difficulties associated with complex surgical procedures and for extending the capabilities of human clinico-surgical interventions [3,4,5,6,7]. Such soft robot-assisted surgery is a rapidly evolving field that allows doctors to perform a variety of minimally invasive procedures with high precision, flexibility and control [8]. Scaling down these devices to micron scale, tiny robots, unlike their large robotic counterparts, can potentially navigate throughout the complex human body, operate in many hard-to-reach tissue locations and hence target many specific health problems [9,10,11]. Therefore, micro/nanorobotics design and materials choice is also seeing a shift from rigid/hard to flexible/soft robots, the latter being more compliant to pass/squeeze through biological systems [12]. Miniaturized soft components with viscoelasticity that match with biological cells are developed as joints, hinges, sensors, actuators and reservoirs to create soft and pliable micro/nanorobots. Often, biological cell machinery is used as micro-engines to drive such pliable synthetic carriers developed via organic structures such as soft polymers and supramolecular ensembles. Any system developed for TDD must work at small scales, where its propulsion occurs at low Reynold’s number. For comparison, it would be like moving in a highly viscous liquid at macro scale like honey. Interestingly, nature has developed motile cells over millions of years for efficient actuation and motion in low Reynolds number regime [13]. In nature, for propulsion at the microscale in low Reynolds number regime, a very versatile micro motor operated flagellum is found in microswimmers like in bacteria, microalgae, spermatozoa and so forth [14,15,16]. Learning from nature, we can directly couple biological microswimmers like bacteria, sperm and algae to propel tiny synthetic robots, leading to this special class of micro/nanorobots called as biohybrid microrobots (schematic Figure 1 and Table 1). When the cargo is drug loaded nanoparticles, such a system is designated as nanorobot [17]. Various approaches which use different motile microorganisms [14,18,19,20,21,22] or contractile cells [13,23,24] as actuators of biohybrid micromotors have been suggested in the literature. The advantage of using certain biological cells is the on-board power source to propel the robots, harnessing the energy from surrounding non-toxic medium.

The avid development of biohybrid microrobots harnessing biological power sources for physiologically compatible nano/microdevices has recently caught the attention of the international research community that is looking for a solution for the actuation and locomotion on the microscale. Any drug-delivery microrobots need to be powered and operated in a physiologically compatible manner. Biological cells such as bacteria have inbuilt stimuli responsive systems against low oxygen (hypoxia), temperature (thermotaxis), magnetic field (magnetotaxis), pH (chemotaxis), glucose (glucotaxis), whereas microalgae show phototaxis and so forth [39]. In fact, the strain MC-1 of the magnetotactic bacterium *Magnetococcus marinus* has been successfully shown to sense the oxygen depleted hypoxic regions within the center of a tumor. It offers the advantage to deliver the drug loaded nanoliposomes directly into the hard-to-reach necrotic tumor core in presence of an external magnetic field [40]. The MC-1 cells contain a chain of magnetic iron-oxide nanocrystals, which were used for directionality in presence of external magnetic resonance imaging (MRI) coils to guide the MC-1 cells towards the hypoxic region via unique magneto-aerotaxis of TDD using micro/nanorobots. Our lab also introduced a bacteria-driven microswimmer lately that combines the sensing capabilities of bacteria for active locomotion with the desirable encapsulation. This biohybrid microsystem shows mammalian cell like viscoelastic properties of a soft double-micelle microemulsion for active transport and delivery of cargo (e.g., imaging agents, genes and drugs) to live macrophages and cancer cells [9,41]. It is beneficial to couple biological propulsion methods with magnetic loads because of the advantages in tracking and the controllability. It has been demonstrated that magnetic resonance imaging (MRI) can be used to perform the dual function of tracking and controlling magnetic micro-/nanoparticles inside living tissue [42,43,44,45]. Further, magnetic load by itself can be therapeutic [46]. However, it is still best to remove any magnetic particles from the bloodstream/body after their intended use to avoid any unnecessary complications [47,48]. On the other hand, effects of long-term biotransformation of magnetic nanoparticles in the living tissue is also being studied [49,50].

## 2. Concept of Spermbots with Undulatory Locomotion

**Figure 2:**
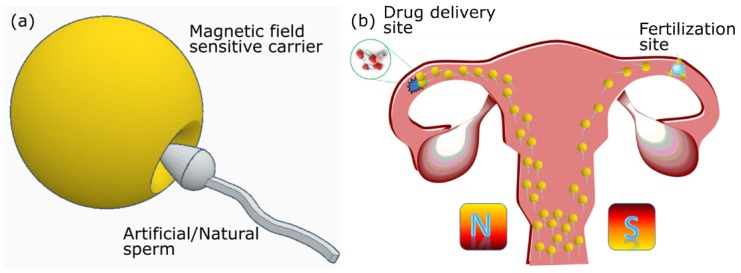
Futurist development of spermbots for target drug delivery and assisted fertilizations. Taking advantage of external guidance and actuation, several spermbots can be actuated towards a specific target, either for drug delivery or for assisted fertilization.

Among microswimmers, sperm cells are known to perform snake like undulatory locomotion for the swimming cells [51]. Such a biohybrid microrobot that uses spermatozoa for propulsion is called as spermbots (Figure 1). There are certain conditions that any drug delivery system, especially the biohybrid systems like the spermbots based micro-/nanorobots, should meet to be effective—(i) Biocompatibility–Such systems should not trigger the immune system and not produce any unwanted complications. (ii) Control mechanisms–The ability to guide/direct the spermbot to a highly targeted location. (iii) Efficient locomotion in the low Reynolds number regime–Considering that locomotion under low Reynolds number is difficult; the spermbot should show time- and energy-efficiency in locomotion when propelled by the cell itself. (iv) Drug carrying capability–Ultimately, the spermbot should be able to do what it is intended for–carry and deliver the drug to the targeted site. Various applications of sperm driven micro-bio-robots in the field of biomedical research areas are emerging that aim at achieving locomotion on the microscale such as drug delivery and single cell manipulation [31,52,53,54,55] (Figure 2). Taking cue from this impressive structure, there has been considerable interest in developing artificial structures mimicking the motion of these flagella using external magnetic fields [20,25,56].

## 3. Creating Biohybrid and Synthetic Spermbots—Technical Challenges and Solutions

**Figure 3:**
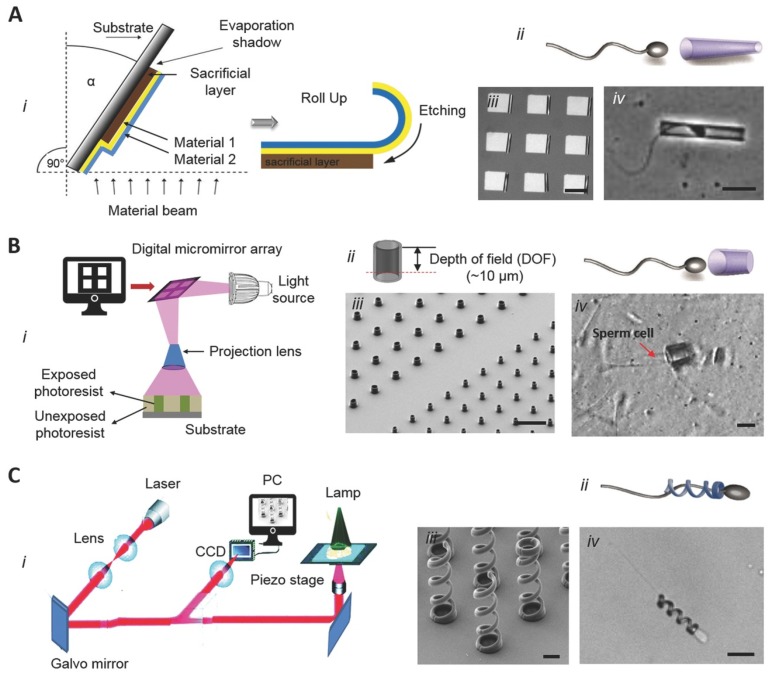
Fabrication routes for tubular (**A**,**B**) and helical (**C**) spermbots. A-(i) A schematic of the rolled-up nanotechnology to fabricate nanomembranes into microtubes. A-(ii) The concept of the tubular spermbots A-(iii) An array of 50 µm-long microtubes. Scale bar 50 µm. A-(iv) An optical image of a tubular spermbot. Scale bar 20 µm. B-(i) The fabrication route for laser-written SU8 microtubes. B-(ii) The control of microtubes length B-(iii) A scanning electron microscopy image of an array of laser-written microtubes. Scale bar 40 µm. B-(iv) The SU8 tube with a trapped spermatozoon. The red arrow indicates the sperm tail. Scale bar 10 µm. C-(i) Fabrication route of 3D nanoprinted helices by two-photon absorption lithography. C-(ii) The concept of helical spermbots to transport immotile sperm cells. C-(iii) A scanning electron microscopy image of the fabricated helices. Scale bar 2 µm. C-(iv) A helical spermbot that is carrying a bovine sperm cell. Scale bar 10 µm. (Reprinted from Reference [22] with permission from John Wiley & Sons, Inc.).

Creating spermbots requires coupling sperm cells with a load. Researchers [55,57,58] have used magnetic nanoparticles coupled with sperm cells for drug delivery and tracking cells in-vivo. However, nanoparticle toxicity is still a contested topic [59,60]. Therefore, the pioneer group for spermbots [27] decided to instead use microtubes to encapsulate the sperm cells. In fact, they were the first group to use real sperm cells to propel a microrobot. The microtubes used are fabricated utilizing microfabrication techniques by rolled-up nanotechnology on photoresist [61,62]. The method followed by the Dresden group, as described in References [22,27,63], is as follows (Figure 3)—A photoresist structure (sacrificial layer) is patterned on a glass slide as squares with 50 µm each dimensions. On these patterns, nanometer thick layers of two different metals are deposited via electron-beam evaporation at an angle. It is stated that the difference in deposition rates and the tilt angle creates a strained bilayer. When the sacrificial layer is dissolved, this strained bilayer naturally rolls into microtubes of 50 µm length and nanometer-scale thin walls. When these microtubes, with the diameter just slightly bigger than the head of the sperm, are immersed in a spermatozoa solution, the sperm cells enter the microtubes, get trapped and start propelling the microtubes along with them. However, this coupling is purely physical and random too. As such, the coupling efficiency is not too high. To increase, the efficiency of coupling, specific molecular binders can be used. There are several biomolecules that can be used to bind the sperm cells to the inner tube surface [64,65,66,67,68,69,70,71,72]. In order to functionalize the inner surface of the microtubes for better entrapment of sperm cells, two attachment methods are utilized—surface linker chemistry and microcontact printing technology. While both the methods improve the trapping of sperm cells, the coupling still relies on random events and is not controlled. Therefore, It will be helpful to develop a method that attaches previously selected single sperm cells to the microtubes in a controlled manner [73].

In the case of artificial spermbots, they can be fabricated using two photon lithography (Nanoscribe GmbH, Eggenstein-Leopoldshafen, Germany). When a photosensitive polymer is coated on a substrate and exposed to a pulse laser of appropriate wavelength, duration and intensity, it gets locally cured due to two photon absorption. When the process is repeated in 3D space while controlled by a computer, it can generate 3D structures with very high resolutions (100 nm) [74]. For the purpose of mimicking the screw like motion of the flagella, microhelices corresponding to the size of sperm cells can be printed (Figure 3). To make these artificial flagella responsive to magnetic fields, they are coated with a magnetic material like Nickel or Iron. To ensure biocompatibility, Titanium layer may also be coated. Such magnetic microhelices demonstrate a screw-like forward/backward motion under rotating magnetic fields. After their release from the substrate, they are transferred to a chamber containing sperm cell solution for coupling. Unlike random coupling in case of spermbots, in this case, the coupling is manual. The microhelices are magnetically driven to an immotile sperm and captured tail-first [30]. With the advent of bottom-up self-assembly, 3D and 4D bioprinting [75,76,77,78], it may be possible to print artificial spermbots in one step in the future.

## 4. Spermbot Assisted Targeted Delivery—Proof of Concept Examples with Assisted Fertilization and Drug Delivery

**Figure 4:**
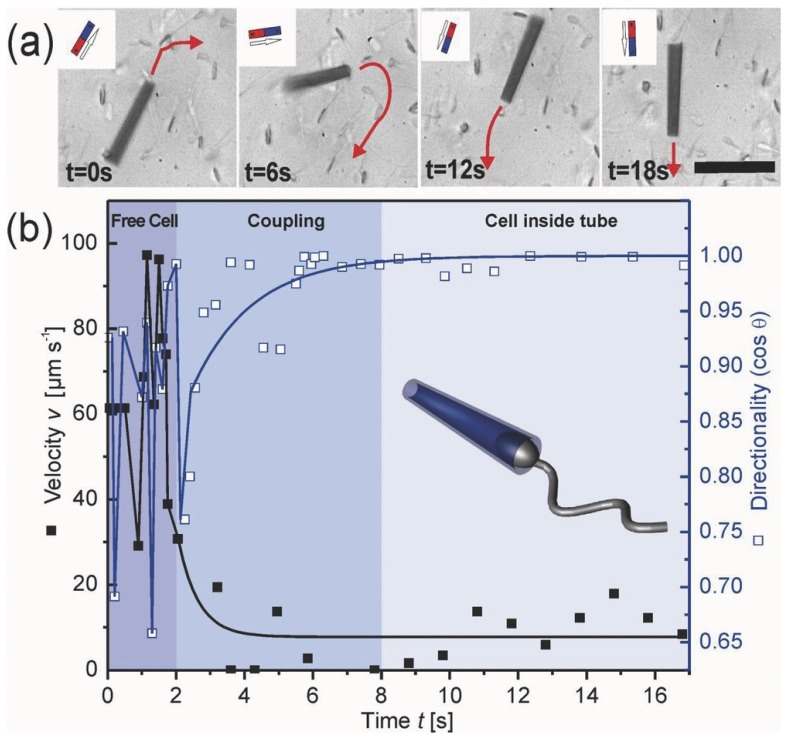
(**a**) Control of magnetic microtube containing a trapped sperm cell by external permanent magnet. Red arrows show moving direction. Scale bar 50 μm. (**b**) Speed (left y-axis, black dots) and directionality (right y-axis, white squares) over the time of coupling process of the sperm cell with the microtube into a spermbot. (Reprinted from Reference [27] with permission from John Wiley & Sons, Inc.).

As mentioned, among microswimmers, sperm cells belong to the fast swimming cells [51]. Spermbots and in general biological motors, are good sources for actuation due to the fact that they do not require toxic media or fuel used like for the chemically powered micromotors for harnessing energy [21,79,80]. In fact, researchers [81] have also designed a metabolic pathway which uses glucose, which is non-toxic, as fuel for sperm-powered nanorobots. They are also generally biocompatible, self-propelled and highly efficient [63]. When coupled with a magnetic material, the spermbots can be directed and the direction of their motion can be controlled very well using external magnetic fields [27,82] (Figure 4). Optical microscopes could also be used for a closed loop guidance of spermbots where line of sight is maintained. While it is expected that the coupling of spermatozoa with a load would slow it down, the spermbot is still very promising among its peers. However, there are some interesting studies which show an improvement in propulsion speed with the addition of load in some kinds of microswimmer experiments, which may help the spermbot community in the future [83,84,85].

**Figure 5:**
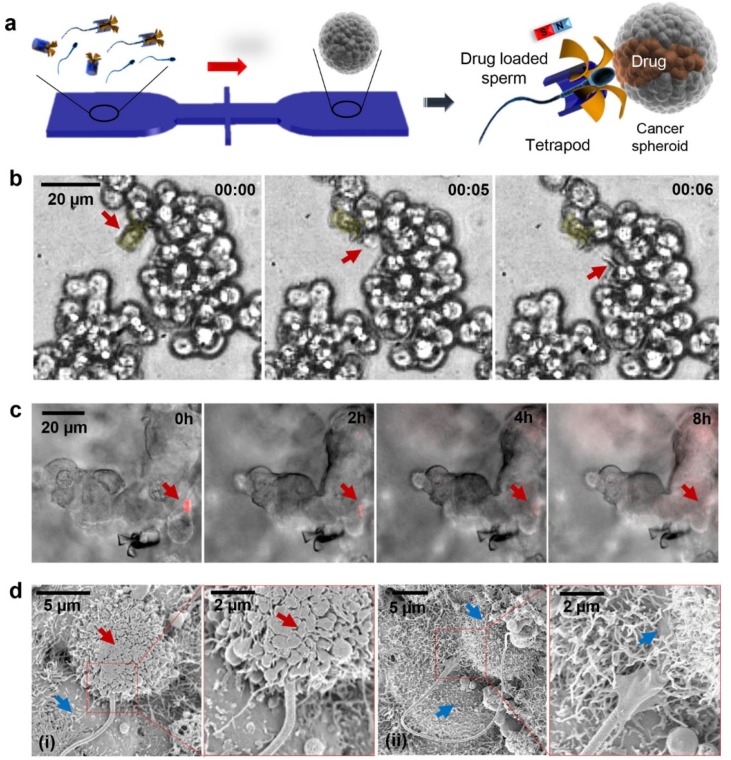
(**a**) Schematic of the microfluidic chip for drug-loaded sperm transport and delivery. (**b**) Image sequence of the sperm release process when the arms hit HeLa cells. Time lapse in min:s. Red arrows point at the sperm head. (**c**) DOX-HCl distribution in a HeLa spheroid with overlaid z-stack images of the fluorescence channel (20 images with a stack separation distance of 2 μm). Red arrows point at the sperm head. (**d**) Scanning electron microscopy (SEM) images showing the sperm–HeLa cell fusion. (i) Cell fusion with the DOX-HCl-loaded sperm; (ii) cell fusion with an unloaded sperm. Red arrows point at a cell in apoptosis and the blue arrows point at live cells. (Reprinted from Reference [31] with permission from ACS Publications).

Sperm driven microrobots have been specifically sought for their TDD potential into the female reproductive tract [31,86]. The spermbots are also considered to be safer than microalgae/bacteria driven robots since they neither express pathogenic proteins nor do they proliferate into biofilms or undesirable colonies. Moreover, sperm cells could be considered as on demand biological cells, which could be considered to deliver to or depart from diseased sites. Exploiting the load carrying capability of spermbots, traceable load could be loaded which may tremendously assist into accurate in vivo tracking. Therefore, the spermbot could additionally serve as explorative device for studying the natural pathways of spermatozoa inside the reproductive systems and neighboring organ [51]. This could lead to a better understanding of obstacles faced by the spermatozoa in the female reproductive tract and eventually, could help in understanding associated infertility. Spermbots have also been proposed for the treatment of cervical cancer [31]. Traditionally, cervical cancer is either treated with invasive surgeries or with chemotherapy. Each comes with its own set of side-effects and disadvantages. As the sperm is already adapted to swimming in this environment, they could act as carriers for TDD. Since sperm naturally fuses with other cells, they could also potentially release drugs directly into the intended targeted cells [87,88] (Figure 5). 

Another specific potential of the sperm-driven micro-biorobot is described [51] that might have impact on the development of assisted reproductive technologies. It is stated that the success rate of state-of-the-art assisted reproduction techniques is still low. These techniques involve the removal of the oocyte from the body, fertilization in the petri dish, cultivation of embryos and reimplantation of the embryo into the uterus. This is where the spermbots can be helpful in bypassing these lengthy and cost associated steps and by guiding the spermbots to the target oocytes inside the human body itself. Schmidt et al. [27,82] used a 50 µm long microtube with iron membrane to encapsulate bovine spermatozoa. The flagellum of the sperm cell serves to propel the microtube forward while the iron membrane is used to steer using untethered magnetic fields. Further, electromagnetic coils with feedback from optical microscope can be used for closed loop control of the spermbots for the targeted delivery to a selected reference point. Schmidt et al. [27] also studied the effect of microtube radius, extent of sperm cell penetration inside the microtube and temperature on the speed of spermbots. With an increase in microtube radius, the extent of spermatozoa penetration also increased. It was found that spermbots with higher penetration percentage have decreased speed because of increased confinement of the flagella. An increase in speed with increase in temperature was also observed. It is reported that, in general, the speed of spermbots is considerably decreased to around 10% of the speed of initial spermatozoa speed. In this case, the coupling of spermatozoa and microtubes also occurs randomly which causes low coupling efficiency. To improve the performance, the same group [73] proposes shortening the length of the microtube to 20 µm from 50 µm. The average speed of the spermbots goes up from about 20% bodylength per second to about 65% bodylength per second. To further enhance the performance, binding the sperm cells to the hollow space of microtubes using Fibronectin (Fn) protein and adding caffeine to the environment to boost the motility of the cells was proposed. As discussed above, there are several biomolecules which can be used to bind the sperm cells to the inner tube surface [64,65,66,67,68,69,70,71,72]. Because the microtube is ferromagnetic, its orientation can be controlled and maintained using an external magnetic field–like a compass needle. Therefore, while the propulsion of an uncoupled sperm cell is random, the direction of propulsion of a coupled spermbot can be highly controlled using an external magnetic field generated either by permanent magnets or by electromagnets.

### Artificial Spermbots

One of the main causes of infertility in men is sometimes called low sperm motility, a condition where the sperm is healthy but unable to swim effectively to make it to an egg for fertilization. Among the couples who struggle to have a baby, the male partner plays a role in the infertility about 40 percent of the time according to the American Society for Reproductive Medicine [89]. Techniques like artificial insemination or in vitro fertilization (IVF) can help but they tend to be not very reliable or complex and expensive [51]. Researchers have come up with a possible solution to help sperm to swim more quickly and effectively with the motorized ‘spermbot.’ Taking cue from the spermatozoa flagella itself, researchers have prepared a metallic helix that can wrap around the flagella of the spermatozoa and propel it using external magnetic field [30]. Once the sperm has entered the egg, the metal casing can reverse direction to slip-off the spermbot with the help of the externally controlled magnetic field. 

**Figure 6:**
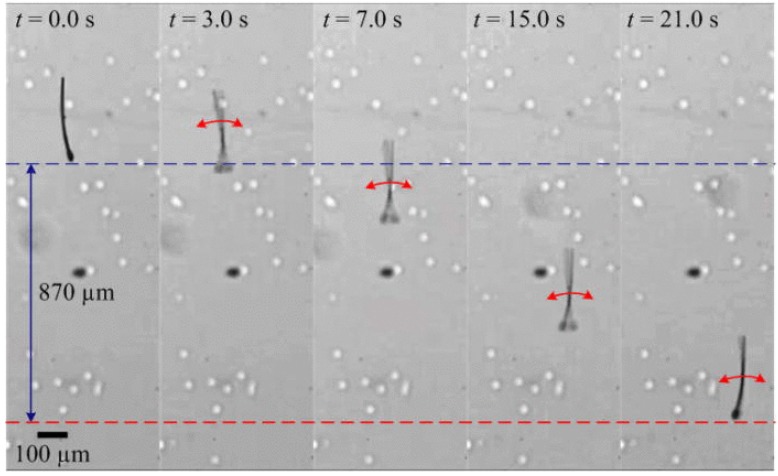
Another kind of Spermbot (MagnetoSperm) moving under the influence of the oscillating (25 Hz) weak magnetic fields (~5 mT). At t = 1 s, oscillating weak magnetic fields are applied which allows MagnetoSperm to swim at a speed of 53 μm/s. (Reprinted from Reference [28] with permission from AIP Publishing).

Researchers [28] have also tried to mimic the entire sperm cell with its head and tail for propulsion using magnetic fields (Figure 6). By employing in-plane oscillating fields, the researchers have shown that such robots reach speeds up to 50% body length per second, which is comparable to the speeds achieved by sperm cells attached to microtubes [73]. The fabrication was done in two steps-the head, neck and tail structures are defined from an SU-8 polymer using photolithography. Then, a 200 nm thick cobalt-nickel layer (Co_80_Ni_20_) is patterned on the head by lift-off. It has also been shown that fully artificial robotic sperm can be fabricated in a single fabrication step using electrospinning [90]. A syringe pump injects the polymer solution of polystyrene in dimethyl formamide (DMF) and iron-oxide nanoparticles at controlled flow rate. High electric potential draws the polymer solution towards the grounded collector. Robotic sperms are collected with the iron-oxide nanoparticles contained within their beads. Such robotic sperms are actuated by applying appropriate magnetic torques on the robotic sperm heads which propagates travelling waves along its flexible tail. Out-of-plane wobbling of the head results in helical wave propagation along the flagellum, whereas in-plane wobbling achieves planar wave propagation. Controllable switching between planar and helical flagellar propulsion has also been shown [91]. Modifying the same process, artificial robotic sperms with two collinear, unequal and opposite tails have also been fabricated which are able to propel back and forth in bi-direction without a U-turn trajectory [92].

## 5. Challenges

Spermbots are not without their own disadvantages in the female reproductive tract because of the hostile environment and natural defense mechanisms. About 30 min after entering the body, <1% of living sperm cells remain in the female reproductive tract due to vaginal flowback, the acidic pH and phagocytosis by leukocytes [51,93]. However, with the microtube enveloping the sperm cell in the spermbot, it could be equipped in ways to prevent the leukocytosis in the same fashion that bacterial pathogens are able to overcome the phagocytic engulfment and killing by appropriate blocking methods [63].

There are several challenges that need to be addressed before spermbots can truly be translated into medical applications:

1. Sperm cells do not all have the same motility. Their motility varies from individual to individual and from cell to cell even from the same individual. This is a cause for concern because we need sperm cells that are highly motile, to be as efficient as possible for actuation. While there is considerable interest in this particular field [94,95,96,97,98,99,100,101,102,103,104], a standardized method and protocol is highly crucial and is the need of the hour.
2. The load/microtubes by itself should not be toxic and should be able to pass through any barriers that it may encounter on the way to the targeted site.
3. The attachment of load or microtubes to the sperm cell is random and a very low-yielding process [27]. Therefore, robust methods to increase this coupling efficiency are needed [38,73]. Electrostatic-based self-assembly helps in partial coating of all sperm heads with magnetic nanoparticle aggregates [38].
4. While the sperm cell by itself is highly biocompatible, there is a chance that harmful microbes could attach themselves to it and render it useless for medical use. To avoid this, antibacterial agents must be applied to the inside of the microtubes to protect the sperm cells.
5. While magnetic fields are very good in directing and orienting the motion of spermbots, other good tactic behavior/control mechanisms also have to be explored. Spermatozoa respond to a variety of stimuli, such as chemotaxis, thermotaxis, thigmotaxis or rheotaxis [105,106,107,108].
6. In order to ensure accurate site-targeting, an appropriate tracking technology is necessary for imaging and guiding of the spermbots. Photoacoustic, Ultrasound and magnetic resonance imaging (MRI) techniques are worth mentioning here as each comes with its own advantages and promises [109,110,111,112,113,114,115,116].
7. There must be an easy-to-control strategy to help release the drug at the intended site once the spermbots reaches there.
8. There may be other application specific challenges. For example, in the case of fertilization with spermbots, we also need to select only the most fertile sperm cells.

## 6. Conclusions and Outlook

Spermbots, while very promising, have some challenges to overcome as described above. The main weakness is the low sperm cell/load coupling efficiency, the loss of actuation speed of spermbots compared to free sperm cells and the imaging and tracking techniques. Therefore, interaction modelling are fundamental challenges for these biologically inspired artificial microrobots.

**Figure 7:**
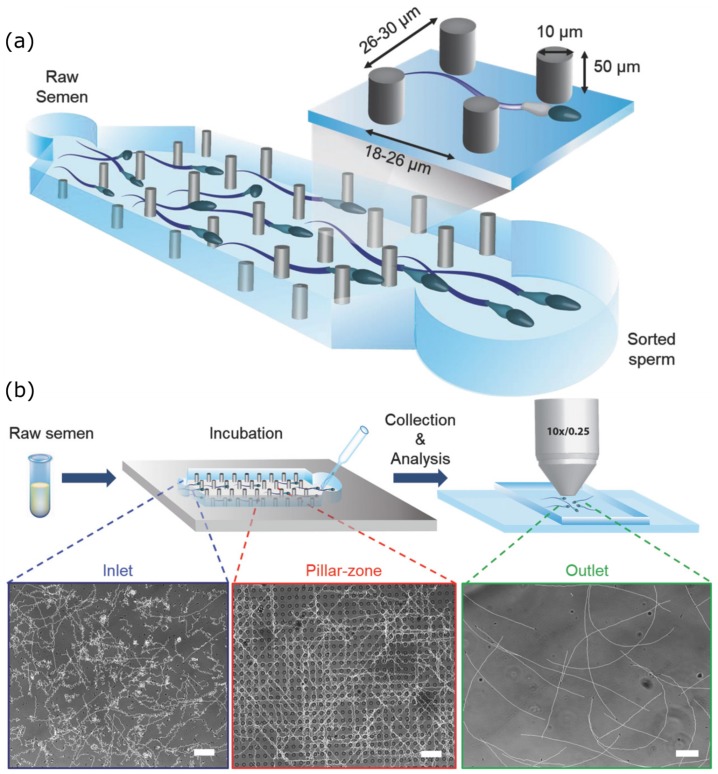
(**a**) Schematic of sorting array device used in SPARTAN. (**b**) Schematic illustrations of the sperm sorting process—semen is initially introduced into the device, allowed to incubate and then output sperm are recovered and analyzed. Microscopy images of sperm trajectories (scale bars: 50 µm). (Reprinted from Reference [104] with permission from John Wiley & Sons, Inc.).

An example of advances to overcome some of these challenges is the Simple Periodic ARray for Trapping and isolation (SPARTAN) [104] (Figure 7), a microfluidic sperm-sorting device. In this process, a simple periodic array device is used to sort out individual sperm cells. Semen is introduced in the device and allowed to incubate. It is stated that the sperm cells with defected morphology find it difficult to maintain a directional motility. Therefore, when the sperm cells are collected at the end of the array, most of them are highly motile. The percentage of motile sperm at the outlet were observed to be higher (~99%) than that of the raw semen at the device inlet (~60%), achieved within a short assay time of 10 min. With such demonstrable techniques, we now need to come up with a standardized procedure and protocol. 

Once all these challenges are overcome, then the collective behavior of microrobots can be harnessed and truly translated into a clinical application. Swarms of microrobots could show higher propulsion speed than single microrobots. Moreover, a swarm will be able to carry a higher combined load to the targeted area. A swarm is also easier to track in the body due to the bigger tracking signal they can generate. In conclusion, spermbots show very promising traits that could open up exciting new applications in the medical field, especially in the female reproductive tract. The aim of spermbot research community should be to develop spermbot which could operate autonomously in swarms, while still giving the surgeons an option to control them. Any artificial load attached to spermbot has to be biocompatible and/or biodegradable. The spermbot should be able to perform its task efficiently. The directional guiding should be non-toxic and harmless by either several tactic stimuli or external stimuli. The targeting capability should be very precise and accurate, considering the side-effects of drug delivery in an unwanted tissue. Finally, drug release should be efficient and effective. As the understanding of spermbots and their interaction with artificial loads and in-vivo biological matter increases, their potential application areas will also keep expanding.

Further, magnetic helices captured sperm delivery to female egg also has to pass through a long road before entering into real infertility clinics. The human trials of these spermbots will uncover the main doubts and lead to next level of applications in IVF reproductive sciences and artificial inseminations. Caution around potential damage to sperm while capturing it and damage to Ova while delivering the sperm via magnetic spermbot are other major concerns. Design and control can be benefited from established soft lithography for synesthetic part design and usage of established clinical imaging. Significant improvement can be made into capturing of the non-motile sperms or delivery of pluripotent spermatozoa in azoospermia (no sperm) patients using these magnetic helices can be further improved via interaction modeling and robotic path-planning related concepts in simulated reproductive organs (Figure 2). Developing techniques and methods to reach the target disease site (e.g., ovarian tumor site) or site of fertilization into fallopian tube and subsequently retrieving back the synthetic robotic part from in vivo can be achieved via MRI and Ultrasound imaging established in clinics. Further, there will be a long road to fulfill roboethics in context with societal, ethical and religious moral opposition and outlook. 

## Figures and Tables

**Table 1:** Timeline for biohybrid sperm development detailing the motor type used and the load carried, if any (Further details can be found in schematic time line in Figure 1).

Year	Authors	Motor Type	Load Type	Reference
2005	Dreyfus et al.	Artificial flagella	Red blood cells	[25]
2005	Weibel et al.	Algae	Polystyrene beads	[26]
2013	Magdanz, Sanchez & Schmidt	Spermatozoa	Magnetic microtubes	[27]
2014	Khalil et al.	Sperm shaped synthetic magnetic microbot	-	[28,29]
2016	Medina-Sanchez et al.	Magnetic microhelices	Low motility spermatozoa	[30]
2018	Xu et al.	Spermatozoa	Doxorubicin hydrochloride (Anti-cancer drug)	[31]
2019	Magdanz et al. Khalil et al.	Spermatozoa	-	[32,33,34,35]
2020	Ridzewski et al.	Spermatozoa	Gelatin microtubes	[36]
2020	Xu et al.	Spermatozoa with a streamlined-horned cap	Heparin-loaded liposomes (Anticoagulant)	[37]
2020	Khalil et al.	Spermatozoa	Magnetic nanoparticles	[38]
